# Podiatric intervention in managing the gait related symptoms of Developmental Coordination Disorder (Dyspraxia). A retrospective study

**DOI:** 10.1186/1757-1146-7-S1-A35

**Published:** 2014-04-08

**Authors:** Pamela Hindmoor

**Affiliations:** 1 Durham School of Podiatric Medicine, New College Durham, Durham, DH1 5ES, UK

## Introduction

The current interventions for children with DCD are predominantly provided by the OT and Physiotherapist. There is a lack of research related to podiatric interventions for the symptoms and disability resulting from DCD in children. The Podiatrist commonly undertakes assessment including: balance deficits, examination of a patient's postural control and gait. Although no studies have identified appropriate interventions for deficits in children with Dyspraxia they are important factors that can substantially affect a patient's function and are important areas for study. A thorough examination of the patient's postural alignment, muscle strength and gait pattern will aid a targeted treatment in which specific exercises and orthotics can be prescribed to improve impairments identified but no studies have been performed to assess their effectiveness in reducing pain or disability.

The main objective of this research was to review the clinical outcomes in a cohort of paediatric patients diagnosed with dyspraxia, attending biomechanics clinic and who had accessed podiatric care for the first time during the course of their condition.

## Design

This research employed a retrospective study design and was conducted in a biomechanics clinical setting in the UK. This study includes data from a cohort of patients referred during 2011/12 and 2012/13 using convenience sampling. The clinical assessments used during this screening programme were based on validated and previously published tools such as Foot Posture Index (FPI), Lower Limb Assessment Score (LLAS). Gait characteristics were measured with pressure plate and gait analysis software. Participants data (group n = 21, mean age 14 years) were compare to a control group (n=20, mean age 11 years) who had no diagnosis of dyspraxia.

## Results and conclusions

A pes planus foot posture (mean FPI score = 8) and hypermobile lower limb (mean LLAS score = 11) was observed. Significant differences in gait characteristic were found between the groups (p < 0.05), following orthotic intervention. The results support podiatric assessment and treatment in the rehabilitation of children with DCD. Further research is required to explore qualitative measures (time to fatigue) in children with this disorder to enhance understanding of foot function is this challenging patient group.

